# “Exotic Sensation” or “*Völkisch* Art”? Press Reviews of the *Indisches Ballett Menaka* (Menaka Indian Ballet) on Tour Through Germany, 1936–1938

**DOI:** 10.1007/s00048-023-00362-1

**Published:** 2023-08-03

**Authors:** Isabella Schwaderer

**Affiliations:** Seminar für Religionswissenschaft, Universität Erfurt,

**Keywords:** Knowledge production, India, Indian dance, Racism, National Socialist cultural politics, Wissensproduktion, Indien, Indischer Tanz, Rassismus, Nationalsozialistische Kulturpolitik

## Abstract

The tour of the Indian Ballet Menaka through hundreds of German cities between 1936 and 1938 left a large footprint in the form of theatre reviews. This article focuses on the role of these performances in actualizing a specific knowledge about India that was, firstly, based on the assumption of the consanguinity of Indo-German peoples and, secondly, on a vision of history as a realization of the utopian ideal of cultural regeneration through art. This article thus hopes to articulate the ways in which the unique experience of this Indian theatre event served as an instrument for consolidating a völkisch/racialized perception of art in general, and of music in particular.

## Abstract (de)

Die Tournee des indischen Balletts Menaka durch Hunderte von deutschen Städten zwischen 1936 und 1938 hat reiche Quellen in Form von Theaterkritiken hinterlassen. Dieser Artikel versucht zu klären, welche Rolle die Aufführungen bei der Aktualisierung eines spezifischen Wissens über Indien spielten, das zum einen auf der Annahme einer Blutsverwandtschaft des deutschen und des indischen Volks beruhte und zum anderen auf einer nationalsozialistischen Utopie der kulturellen Erneuerung durch die Kunst. Es soll dargestellt werden, inwiefern die einzigartige Erfahrung dieses indischen Theaterereignisses als Diskursinstrument zur Konsolidierung einer völkischen Wahrnehmung von Kunst im Allgemeinen und vor allen von Musik, diente.

Between 1936 and mid-1938, the choreographer and dancer Leila Roy Sokhey (1899–1947), also known as Madame Menaka, led her dance troupe, consisting of three female and two male dancers, accompanied by a musical ensemble, through a large-scale European tour with approximately 600 to 700 performances (Joshi 1989: 27). They performed in Germany as well as the Netherlands, Switzerland, Austria, Italy, Hungary, and even Estonia. They danced and played almost every evening on different stages in urban centres and in provincial towns, in theatres and cinemas, festival halls, and sanatoria. The press response was enthusiastic. Most theatre critics were delighted and cheered the unique, colourful, and sensual programme of previously unknown music and graceful dances. There were also other reasons for the enthusiasm of the spectators. For instance, a critic from Hagen concluded his article about an “unforgettable evening at the Municipal Theatre” with the following exclamation: “Menaka danced! She ‘celebrated’ the high art of ancient Indian culture. She danced a distant period of strong ethnic character from a land that received its oldest and richest cultural impregnations through the Aryan bloodstream (W.P. 1936).” This quotation was no exception in the polyphonic press response to the Indian evenings. Again and again, journalists referred to the Aryan race of the Indians or their allegedly Indo-Germanic origins. Firstly, this article examines how the press echo related to the cultural-political discourses of the time and how it presupposed a knowledge of India that both philological and non-academic publications in Germany had already created much earlier. Secondly, it illustrates how such knowledge was actualized and reinterpreted within the National Socialists’ racially structured view of humankind and the world.

## Sources and Research Questions

The sources presented here are part of the Menaka Archive, an artistic collection project by Markus Schlaffke (Schlaffke 2022). Beginning in 2018, we systematically located and compiled the reviews of all documented Menaka performances in German regional newspaper archives and in international digital archives as a separate project inside the archive (Schlaffke & Schwaderer 2021: 5–6). In the following, I will focus on selected articles from the tour’s press reviews, especially those that address the kinship of the performers on stage with the audience. They come from a homogeneous corpus of theatre reviews published between 1936 and 1938: around 120 texts, mostly consisting of a two-column text of about 500 words in the elaborate language of the culturally informed, full of references to contemporary cultural debates. All articles refer to the same programme performed (with only minor variations) in each theatre. All the more surprising is the variety of impressions that the critics recorded. However, this text corpus’s fundamental problem quickly becomes evident: We are dealing with mostly “grey” sources, that is, the author(s) are usually not known by name. Only a limited number of texts mention an author—possibly guest contributors. An initial investigation revealed that these individuals were often representatives of the local middle class, that is, directors of music schools, musicologists, and editors-in-chief of various feuilletons. In order to make other voices audible, personal, unpublished material would be a valuable addition. There are also no known personal recordings of the artists themselves in the archive; however, they could allow interesting insights in the future, as could first-hand material from audience members. However, since these types of materials cannot be searched for systematically, the archive is dependent on fortuitous discoveries.

With the subject of this special issue in mind, this text corpus can be helpful in solving the following questions: How can the reception of the Menaka Ballet shed light on the broader cultural knowledge about India in German-speaking Europe? What aspects inherent to this knowledge—for example, orientalist, racist-ethnic, and ideological stereotypes—emerge from the articles, and how did the various groups of people involved in writing and reading them contribute to this? In particular, how do the texts reveal traces of the importance they attached to the assumed notion of *Aryan* or *Indo-Germanic* origins? Finally, what was the role of India as a medium for projecting the highly debated ideologies of this time?

The normative boundaries of belonging and foreignness implicitly expressed in the texts can help to determine the cornerstones of the entire discourse around identity. In order to uncover the ideological dimension of the texts, a cultural semiotic analysis of word fields was used to reconstitute the representation of the Indian dancers as Indians, in the sense of being individuals who belong to a particular group. First, however, this article will briefly outline the particular cultural-political conditions under which these texts were written to identify the most important debates of this period.

## Some Preliminary Remarks on National Socialist Cultural Politics

The strategies used by the Ministry of Popular Enlightenment and Propaganda (*Ministerium für Volksaufklärung und Propaganda*) to force the cultural sector to conform (*Gleichschaltung*) are well documented. Half a year after establishing the ministry, Joseph Goebbels struck a decisive blow against all free artistic creation. The Reich Chamber of Culture (*Reichskulturkammer*) and its subdivisions, an amalgamation of the Ministry of Arts and different art sectors which would remain self-administered, was established by law on 22 September 1933 with the sole responsibility of deciding who qualified as an artist. The door to the Reich Chamber of Culture thus predictably was closed to non-Aryans and non-conformists (Frei & Schmitz 2014). The coordination of artists went hand in hand with that of editors and publishers. By coordinating news offices, permanent press conferences, and many press services with their numerous language regulations the media was able to convey a cohesive Nazi cultural and press policy. Due to countless restrictions and the fact that many newsrooms lacked qualified employees after the anti-Jewish and anti-communist purges of 1933–1935, monotony and boredom soon began to dominate the German cultural and press landscape (Frei & Schmitz 2014; Fröhlich 1974: 354). In this context of cultural desolation, it is not surprising that the performances of the Indian Ballet Menaka attracted a great deal of attention, if not enthusiasm, despite their unconventional programme. The wide ranging press coverage is evidence of both this phenomenon and—as we will see—of a more extensive socio-political discourse in Nazi Germany.

As Frei and Schmitz have discussed, journalism in the Third Reich was a less clear-cut matter than it appears in contemporary historic-political debates. While the regime’s rigorous claim to total control of public opinion was implemented rapidly, there were nevertheless differences in press coverage (Frei & Schmitz 2014: 7). During the Menaka tour, a significant debate ensued about the importance of non-political texts in the culture section of newspapers. The importance accorded by the National Socialists to the cultural section (*feuilleton*) within the press as a whole was made clear by Rainer Schlösser on the occasion of the *First Press Conference on Cultural Politics* on 24 July 1936 in Berlin: “The average German, who until recently was all too often not very politically inclined, and often remains so even today, often reaches the political editorial via the culture section, that is via the feuilleton. For a long time, this has not been recognized (Haacke 1951f: 154).”

It was apparent to those involved that the feuilleton was the ideal place to influence public opinion beyond placatory—and, thus, rather inefficient—propaganda. Art was evaluated according to its propagandistic potential and then used intentionally in the cultural press.

Ernst Krauss (1887–1958), the impresario of the tour, was well aware of this,^1^ often organizing the performances in cooperation with local National Socialist organizations. From Landshut, for instance, one reads the following, dated 23 April 1936: “We are very grateful to the NS cultural community for giving us the rare pleasure of spending more than 2 hours in a completely foreign and mysterious world” (At.: 1936). On 21 April, two days prior, the organization *Kraft durch Freude* (Strength through Joy) had offered tickets at reduced prices; the same happened for the performance in Garmisch, where the critic was somewhat sceptical about the performance. “As I said, the evening was also a little tiring due to the monotony of the orchestra, but it did offer impressions of the rarest foreign culture. And this is also a task of the ‘NS Cultural Community’ [*NS Kulturgemeinde*]” (AH: 1936). In Tübingen, the National Socialist cultural association *Kulturbund* helped host the performance; theatre critics echoed the typical rhetoric of the host community afterwards.

Menaka’s Indian ballet, in spite of its unusual programme, was not only tolerated in Germany in the middle of the Nazi era, but it was also enthusiastically celebrated in both urban centres and provincial towns, on illustrious stages as well as in small venues like health resorts and spas. Interestingly enough, looking at the advertising through different National Socialist cultural organizations, the Indian programme even seemed to fit into the cultural agenda of at least some of the associations that had spun off from Alfred Rosenberg’s Combat League for German Culture (*Kampfbund für Deutsche Kultur*), one of the most active early Nazi organizations. Founded in 1929 with the goal of strengthening allegedly suppressed Aryan artists and eliminating the so-called degenerate ones, the Combat League not only published propagandistic brochures (including reviews of Jewish and modernist musicians), but also funded ideologically-aligned artists and pursued aggressive practices such as the disruption of concerts, threats to performers it disliked, and intimidation of audiences (Bollmus 2006; Gimmel 2008; Walsdorf 2010). After being renamed the *National Socialist Cultural Community* in 1934, Rosenberg’s *Combat League* was gradually absorbed into *Strength through Joy* (Bollmus 2006: 101). Both organizations promoted a diverse range of cultural events, including the Menaka troupe’s performances. Without inhibiting or encouraging public criticism, the National Socialists could—or so it seemed—proceed to realize a cultural programme in line with their views. The ensemble of guidelines and organizational measures with which they sought to bring culture into line ranged from the recommendation or financial support of certain artists to the prohibition of other artists’ work. It has been aptly described as a “guided intellectual life” (*gelenktes Geistesleben*) and included bans on performances, coordination (*Gleichschaltung*), censorship, the elimination of critical intellectuals and democrats, the installation of concentration camps and, last but not least, the persecution of Jewish artists (Wilson 2018: 235).

Examining the Menaka tour’s press coverage thus allows for unique insights into how both ideas about dance and knowledge of India were used by the National Socialist ideological apparatus. Even if not academic in nature, the rather unusual corpus of theatre reviews evokes both the debate about what is familiar or one’s own versus what is foreign and about the essence of culture. These arguments were not new at that time and not even specifically National Socialist in themselves, as Walsdorf aptly notes in regard to the debates around folkdance (*Volkstanz*) in the 1930s (Walsdorf 2010: 97). The debates both on Indian art as well as on dance more broadly can be seen as part of a cultural-political proxy war fought for the sake of determining what art should qualify as legitimate in the context of the Third Reich.

The corpus examined here is also unique because each of the texts has the same starting point, namely the Indian Ballet Menaka’s stage performances, which were (almost) identical in every city. Nevertheless, critics wrote utterly different, sometimes contradictory things about the performances, as we shall see. Thus, even when operating in a very closely monitored discursive arena, it seems that this cultural event sparked a wider debate around certain particularly contested issues. These include questions about the meaning of racial (*völkisch*) or racially akin (*arteigen*) art (in particular what the meaning of a racial/*völkisch* culture might be in the first place), the grounds upon which art should be judged, and, beyond this, how the arts might contribute to a social revolution in National Socialist terms.

## A Cultural-Semiotic Approach

For the evaluation of the material outlined above, a cultural-linguistic or cultural-semiotic approach was chosen. This approach identifies patterns or codes and conventions in culture to extract coherent and meaningful messages from an otherwise complex and ambiguous context. The basic assumption here is that, in writing a review of an ‘Indian Evening’, contemporary critics reflect (more or less consciously) their own idea of culture, whether in racial/*völkisch* terms or through other normative lenses. In Juri Lotman’s notion of the semiosphere, different layers of author-reader communication emerge. The extraordinary experience of witnessing the theatre performance seems to have generated a combination of irritation and excitement—or, at least, that is what is reflected in the newspaper articles. The texts also reveal the intersection between political and artistic spheres, as well as the interconnections between artists and spectators, journalists, and readers. Its authors are all urged to position themselves in a complex field of ideological allegiances and perspectives on artistic expression. In this sense, the whole communication system at play can be understood as a *semiosphere*, which, in Lotman’s words, is “immersed in a specific semiotic continuum, [one] which is filled with multi-variant semiotic models situated at a range of hierarchical levels (Lotman 2005 [1984]: 206).” This semiosphere thus allows for a conceptualization of the intersecting discourses that form the background against which the press reviews of the Menaka Ballet can be read.

The use of language in Germany from 1933 to 1945 has been studied from various perspectives, included extensive analyses of organizational and administrative vocabulary from the field of propaganda and rhetoric as well as stylistics. As Kämper has pointed out, however, there is a lack of studies on actor-specific language use and communicative-interactive practices based on larger corpora (Kämper & Schuster 2018: 2). She has herself attempted to fill this gap with a cultural-linguistic approach that seeks to determine the extent to which certain tropes originated from within a broader intellectual context and how they are reflected in their textual production (Kämper 2018b: 10). The press landscape described above biased the authors of the reviews, in the sense that the possible opinions they could express were limited to complying or at least not to expressing anti-government opinions, since they all belonged to an already limited pool of journalists who fit into the social molds created by the state from 1933 to 1945.

This article thus focuses on specific terms representing turning points in the coordination of cultural life from 1936 to 1938. In these three years, various actors formed an interpretative scaffolding that ultimately resulted in the total absorption of public expression into the ideological framework of the National Socialists. Journalists had to comply with a racist and ideological order that included being part of the People’s Community (*Volksgemeinschaft*) and acting in accordance with the strictures of a totalitarian dictatorship intent on protecting its political-ideological agenda. Therefore, this study does not claim to represent the entirety of the German discourse; on the contrary, it is (necessarily) limited to a section of “conformists” (Kämper 2018b: 10). The critic’s encounter with the artists and their subsequent reflections on the work they observed thus offers an excellent template for a cultural semiotic analysis, since this small encounter illuminates, as if under a magnifying glass, precisely what determined the debates around art: the confrontation with the Other, the attempt at self-definition and the search for the *self* in the *foreign*, that is, the questioning of Indians’ racial kinship with Germans. Such research reveals the boundaries of societal debate in a format that, while at the margins of political arguments, reflects the rapidly growing ubiquity of such conversations in German society.

Keeping in mind the severe restrictions on freedom of expression in the coordinated cultural sector and in the media, this article identifies different worldview schemata inherent to the divergent discourses that existed around a highly controversial cultural concept, namely that of racialized identity. The theatre reviews develop different intertwining discourses and exchanges between actors and society. In other words, actors are individually and collectively involved in social interactions and together integrate different educational and experiential horizons. Nevertheless, a common denominator is found in how they locate their views and themselves when confronted with the then vital question of how to express self-affirmation.

## Indians and Germans: Strangers or Distant Relatives?

The spectators of the ‘Indian Evening’ likely arrived at the theatres with quite precise preconceptions. The press statements had promised both exotic fantasies and an ancient art form that hailed from the temples, and this engendered a broad spectrum of expectations. Both critics and the public had few real-world experiences or prior encounters with contemporary Indian art, let alone with its contemporary dance. As such, they based their contextualization on pre-existing knowledge derived from the nineteenth century. Spectators consequently translated the events on stage, which were very unfamiliar to them, into a vastly different cultural context shaped by their bourgeois educational upbringing—which one can assume, based on who had the means and distinct taste to attend theatrical performances of this kind. However, the reviews address the emotionally charged and contested issues of religion, culture, cultural criticism, and national affiliation. They connect significant signposts marking the transition between the *inside* and *outside* of a specific discursive space and frequently point at the intersection of the different realms forming the semiosphere. The inclusion and exclusion processes also lay bare not only the intellectual juxtapositions of art and politics, history and utopia, but also origin myths and idealized communities.

To elaborate on this, I propose a classification of three different aspects of the vision of Indian culture and its relation to the idea of the German “us” constructed by the respective authors: an *exoticized India*, or a perceived *foreign in the foreign*; *Indians as (distant) relatives*, which points at the *familiar in the foreign*; and on the one hand *India as part of the Romantic vision of families of peoples, *and, on the other hand, to a supposed *racial consanguinity*. As the following examples show, more than one category can be addressed in the same text.

‘Exotic’ Indian dance initially found its audience in Germany as a vehicle for promoting colonial goods and as part of the so-called peoples’ exhibitions (*Völkerschauen*), the Hagenbeck brothers’ flourishing entertainment and souvenir business. Troupes of female temple dancers were recruited from the temples of Tanjore, the cultural centre of southern India. The dancers were then presented together with other entertainers, such as jugglers, animal trainers, sword swallowers (Zubrzycki 2018: 191). These and similar exhibitions presented colonialism and popular science, such as biology and anthropology, as mass spectacles (Reichardt 2008). Moreover, popular media such as novels and films contained a whole range of exotic, orientalizing, and colonial fantasies. A prominent example was the bestselling novel *The Indian Tomb* (2016), written by Thea von Harbou (1888–1954) in 1918 and adapted for film several times in subsequent years. In Richard Eichberg’s film *The Tiger of Eshnapur* (1938), the Menaka Ballet appears briefly after the troupe was hired for a film shoot in Berlin—the only footage of the troupe that has been preserved. It features segments of a dance drama *Krishna-Leela* that was also performed as part of their events on German stages. As extras in a monumental production, the dancers and musicians illustrate a colonial fantasy driven by a desire for conquest (Struck 2010). The presence of *real* Indian artists among the obviously European performers wearing brown makeup to *look* Indian marks a break in the pop-cultural appropriation of the exotic (Schlaffke 2022: 84–110). Both movie and theatre critics enhanced the ‘exotic’ features of the artists, serving as an example of the racialized gaze (Dreesbach 2005; Lewerenz 2006; Ciarlo 2011). Eichberg’s film thus depicts the multifaceted and self-contradicting nature of the German understanding of the Indian foreigner as one undergirded by a complex interplay involving between love, power, and jealousy in its many different forms. The Menaka Ballet here embodies the *foreign in the foreign* on screen, and it serves as a distorting mirror for the projection of identity.

However, the subject of this paper focusses on the different variations of another form of the experience of foreignness, one in which *identity reveals itself in alterity*. In the Romantic understanding of the origins of cultures, divisions emerged from the biblical table of peoples (Gen 10,2). From the list of mythical names referring to primordial peoples, only that of the *Semites* was transferred into a division of peoples living in a world increasingly structured by linguistic considerations (Arvidsson 2006: 13–14). India as part of the Romantic vision of families of peoples started from a linguistic category. The Danish geographer Conrad Malte-Brun was the first to use the term “indo-germaniques” (Malte-Brun 1810: 577) for languages existing between the two geographical extremes of the language family: India in the southeast and Iceland in the northwest. In German and English-speaking areas, the designation *Indo-Germanic* prevailed over *Indo-European*, which was more common in France. Parallel to Indo-Germanic studies as a linguistic discipline, Indo-Germanic archaeology (*Indogermanische Altertumskunde*) developed into a discipline that investigated the “material and immaterial (spiritual) culture of the Indo-Germanic speaker community” (Schmitt & Häusler 2000: 384). Similarly, the term *Aryan* developed from an ancient Persian self-designation into a designation of a language group, the Indo-Iranian languages, also called *Indo-Aryan*, or merely *Aryan*. The idea of kinship was later on extended from languages to cultural features and religion (Arvidsson 2006: 43), establishing a link between linguistic similarity and notions of consanguinity. A clear shift in meaning resulted from the adoption of the terms *Indo-Germanic* and *Aryan* in popular historiographical and philosophical works as early as the nineteenth century. A popular notion of India was constantly repeated—the notion of some primordial kinship between speakers of a related language. Such tendencies were primarily rooted in the narrative of an alleged continuity between *Aryan/Indo-Germanic* culture, one which was nourished via the popularization and dissemination of Indological philosophical writings and semi-academic prose by figures such as Paul Deussen (1845–1919), professor for the history of philosophy at the University of Kiel and founder of the Schopenhauer Society, Leopold von Schroeder (1851–1920), professor for Old Indian philosophy and archaeology in Vienna, and Houston Stewart Chamberlain (1855–1925), an influential member of the Wagner family and prolific writer. They belonged to learned circles that shared an interest in the religious and philosophical worldviews of Ancient Indians, following Arthur Schopenhauer and Richard Wagner in their adaptation of what they thought was an *Indian* or *Buddhist *religion or philosophy. From here, they formed a counter-culture with antisemitic leanings that used ‘Indian’ clichés as a starting point for a reorientation of Christianity under national auspices (Schwaderer 2022; Schwaderer forthcoming). This narrative, which thought positively of Indians, was anchored in a bourgeois erudition that was not always congruent with the crude contemporary racial discourse of National Socialism on the question of Indians’ relation to Germans (Roy 2017).

From cultural proximity it was only one more step to a biologistic interpretation. Chamberlain promoted the ideas of Joseph Arthur de Gobineau (1853–1855), at first through reviews and eventually through his bestselling book *Grundlagen des neunzehnten Jahrhunderts *(1899), which influenced Alfred Rosenberg’s *Mythus des zwanzigsten Jahrhunderts *(1930) (Lobenstein-Reichmann 2008: 436). Chamberlain’s historical speculations departed from the assumption that Western civilization had arisen under the strong influence of the Germanic people and created a metanarrative interweaved with the concept of Indo-Germanic linguistic affinity and a considerable amount of social Darwinism. Here, and in his later writings, he elaborated on the cultural-religious ideas of Deussen, von Schroeder and other thinkers invested in a paradigm of ‘Aryan Religion’ (Bermbach 2015).

It is almost impossible to reconstruct a clear and traceable understanding of what these authors perceived as *Aryan, Indo-Aryan* or *Indo-Germanic* culture and religion and how they were related to more general ideas in the Romantic era. What is obvious is that locating the notion of the origin of cultures in Europe was deemed a precarious and fragile hypothesis. One idea to compensate for this fragility was to relocate the origin of the Germans to India (Schwab 1950; von See 1970; for the discussion see Grünendahl 2012). Around the turn of the century, German thinkers under the influence of Chamberlain’s historical scenario postulated a second *Indian Renaissance* as a German epochal threshold: once in the philosophy of Romanticism in the wake of Herder and Schlegel, and again in Chamberlain’s writings (Gotthelf 1917: 267).^2^ From a clearly National Socialist perspective, Hans K.-F. Günther (1891–1968) then adapted the designation Indo-German in his works *Die nordische Rasse bei den Indogermanen Asiens. Zugleich ein Beitrag zur Frage nach der Urheimat und Rassenherkunft der Indogermanen* (Günther 1934) and *Herkunft und Rassengeschichte der Germanen* (Günther 1935), where he postulated a Nordic origin for the Indo-Germans. Moreover, as Volker Zotz points out, Heinrich Himmler’s propaganda institute, the Forschungsgemeinschaft Deutsches Ahnenerbe [Research Foundation German Ancestral Heritage], and various university institutes elaborated several theories of the German Aryan, but without reaching an overall conclusion—despite recurring references to the Führer principle, race, blood and soil (Zotz 2017: 7). The word *Aryan*, finally, served in National Socialist propaganda as a synonym for “member of the (Nordic) German race” and, *ex negativo*, as a synonym for “non-Jewish” (Schmitz-Berning 2007, s.v. *Arier, arisch*, 54 ff.).

The authors of the texts analysed as part of the coordinated press paradigm adopted, at least partially, a racist worldview of different, hierarchically arranged families of peoples or races (Burleigh & Wippermann 1991; Weingart et al. 2017; Wernsing et al. 2018) and its language and expressions. Interestingly, a unitary ideology from the heterogeneous conglomerate of dominant ideas about “human races” (Wernsing et al. 2018: 23) is difficult to discern. Nevertheless, the reference to an *Aryan family of peoples* and to a division of humanity into different group or *races* fits into a particular mechanism “by which worldview changes into objectivity” (Etzemüller 2015: 8). Etzemüller argues that the allegedly scientific discipline was in fact “a social doctrine in the guise of biology” (ibid.: 10), one which “successfully produced results in a specific time with methods recognized as scientific, which must be understood as pseudoscientific, but which […] had considerable repercussions on the bodies and lives of countless people” (ibid.: 12).

In the case of the Menaka Ballet, the question of whether the people on stage were Aryans was essential, so much so that the artists themselves were aware of it. On 18 February 1935, when plans to tour through Germany were already becoming concrete, Leila Sokhey gave an interview to the Indian newspaper *The Hindu*. In it, she expressed doubts about her acceptance by the German head of state, or, as the article put it:

“When she toured the capitals of Europe two years ago [1931–32, during her first tour through Europe] she was very well received, especially in Berlin, ‘But I do not know if Hitler will tolerate my presence,’ she added smiling, ‘I hear that he has proved to his own satisfaction that Indians are non-Aryans.’” (Anonymous, *The Hindu* 1935).

Even before the artists had arrived in Germany, they too had absorbed this binary classification of Aryan or non-Aryan as accurate and relevant. This relates in part to debates that were then taking place in India, where the press engaged with Nazi racial theories and where, interestingly, it was not so much the idea of a hierarchy of races that drew criticism, but the fact that Hitler did not consider Indians to be members of the Aryan race (Framke 2013: 117–130). Conversely, Heinrich Himmler had a clearly more positive view of India at this time than did Hitler or Rosenberg—probably also with regard to the independence struggle of the Indians against Great Britain^3^—which was nevertheless supported by a majority in the regime from the 1930s onwards (see Delfs 2008: 72 and passim). It is noteworthy that the almost exclusively positive reviews of the ballet in Germany repeatedly emphasized the theme of consanguinity, which is something Sokhey likely never expected from German critics.

The superficial impression of a primordial kinship of some kind between *Indo-Germans* was enough for the troupe to be carried from one venue to the next on a wave of racialized sympathy in Germany. Sokhey stated in a 1936 interview in Dresden that she had never been received with such appreciation as in Germany, or, in the interviewer’s words: “One thing, she said, was particularly remarkable for her: here in Germany, her art resonated everywhere, in the big cities as well as in the provinces, while in other countries there was only genuine interest in the capitals (Dotzler 1936).”^4^ From a financial perspective, Sokhey was undoubtedly correct. Due to overwhelming demand, the tour was extended several times, and guest performances were organized in almost all German theatres. However, beyond the artistic quality of the performances, could there be other reasons for the programme’s success throughout Germany?

## Case Study 1: the Authentic-Genuine Semantic Field

The first semantic field selected is grouped around the idea of something being authentic, or *genuine*. As Kämper points out, *authentic* “is to be interpreted less as a designation than as a figure of thought that brings together corresponding linguistic realizations on a higher level of abstraction” (Kämper 2018b: 15). An examination of this semantic field in the Menaka critiques confirms Kämper’s assumption, namely, the large extent to which this figure of thought is recognizable in linguistic realizations (ibid.). *Authenticity* is a status that results from a collective linguistic-conceptual understanding about the past based on both evaluations of knowledge systems and the linguistic-discursive realization of these evaluations (Kämper 2018a: 28); the normative aspect of the category *authentic* goes back to Romantic classifications of cultural origins (Almeida 2017: 60–64).

The *authentic* is understood here as a normative category within the racist National Socialist worldview. During the regime’s political consolidation, highly emotional slogans were repeated to the point of exhaustion, together with their evaluative epithets, such as the semantic field *echt* (“real”) or *einmalig* (“singular”). The latter denotes a “need for enhancement, if possible, up to the superlative… However, with *echt*, it goes inwards, into the confidential, the *human*, straight to the seam of the heart (Sternberger et al. 1970: 38).”

The authenticity of the costumes and the originality of the orchestra were indeed exceptional, especially as the house orchestra usually accompanied guest performances including dance, in Europe. This was, for instance, the case during Madame Menaka’s first tour, which took her through Europe with her dance partner Nilkanta. It is therefore unsurprising that this unique was emphasized in many announcements, advertisements, and commercials. Nevertheless, several theatre critics placed the concept of uniqueness into a different context. A particularly striking example is an article from the *Bayerische Ostmark/Coburger Nationalzeitung*.^5^ The day after a guest performance in June 1936 in Coburg, Josef Knodt, the lead editor for local affairs, National Socialist politics and general affairs at the *Bayerische Ostmark*, published the following:

“For a healthy people (*Volk*), dance is an expression of its soul. It is thus an important and necessary celebratory form. We therefore place the folk dance alongside the dramatic play of music as an unequivocal form of self-expression. For the Romance and Nordic people, folk dance is second nature. We have always tried to build a bridge from the aesthetic form of folk dance to the stage dance, and in doing so, we have gone through all kinds of variations in the pantomimic, the realistic, the emotional, or the mixed. How wonderfully we have progressed with this is shown in the confrontation between the Viennese and the [*n*-word] exoticism, whose ornaments and symbols still echo in our operettas. An evening at Menaka leads us to reflection. It is not as if we should fall from one extreme to the other, no, we should recognize from this example how a form of expression that has been in use throughout the centuries is, despite or precisely because of its contemporary adaptation, an inherent (*ureigen*) element of a people” (Knodt 1936).

It is worth noting that Knodt took great care to make his theatre review something of a political statement as well. Indeed, he used the performance as an occasion to publish a wider reflection on matters pertinent to both politics and dance theory, something already evident in his review’s opening words: “The question of whether it is worth thinking about stripping dance of its social aura and replacing it with a desire for a national form of expression is answered by a performance such as yesterday’s at the *Landestheater* (Knodt 1936).” Without mentioning the dance performances, the author proceeds to make fundamental statements about the importance of dance, namely as a form of expression for psychological processes. What he introduces here as an anthropological constant is distinguished, depending on the context, into mastery (“the Viennese”) and decadence (the “[*n*-word] exotic”), whereby the aesthetic category of virtuosity is equated with racial integrity. Characteristic of the sharp separation of the “familiar” from the “foreign” are fears of degeneration and scenarios of threat, which are a part of this linguistic pattern familiar from the collapse of the German Empire (Weingart et al. 2017), as studied by Föllmer in the context of the rhetorical trope of health body of a people (*gesunder Volkskörper*) (Föllmer 2001). If there is mention of a “healthy people”, this enhances its fragility and amplifies the threat of elements that could end up making it a “sick” one, via the introduction of elements from (racially) alien forms. The symbols of the “Viennese” lead to the final remark to take the performance of the Menaka as a model, to adapt traditional “forms of expression” to the times, but not to move away from what is inherent to “a people” (note the highly emotional character of the German *ureigen*).

In any case, these ethnographic reflections on the phenomenon of dance are not neutral. They serve to create the category of a “community of people” (*Volksgemeinschaft*) which is inseparable from ideas of inclusion and exclusion, ones which permeate all levels of linguistic action. This is seen in how a dance performance had to appear “foreign” in many respects: the music, the gestures, and the costumes. Interestingly, however, it is not the Indian artists who are excluded here. The linguistic inclusion and exclusion pattern, especially on a semantic-conceptual level, does not refer to the *foreign* Indians. On the contrary, the performance is set as a normative example of “how a form of expression that has been valid through the centuries is, despite or precisely with its contemporary adaptation, an inherent element of a people” (Knodt 1936).

As such, the exclusionary social construction underlying not only Knodt’s review, but that of countless others of the Menaka Ballet’s performances, only becomes clear upon closer examination. The difficulty in immediately recognizing the underlying dichotomy they promote and who its proper referents are lies in the fact that the excluded segment of the binary is not directly addressed, but only hinted at briefly and abstractly at that (“the [*n*-word] exotic”). Aside from this instance, it is almost deliberately concealed. It has long been known that the potential for violence inherent to racist discourses lies in linguistic non-representation, without necessarily having to resort to aggressive attributions—such as “the others make us sick”. The reference to “healthy” people is enough to turn the potential threat into a “real” scenario for the reader, who is well aware of the surrounding discourse.

The remark about the *Evening at Menaka*, which “brings the viewer to his senses”, or which brought something essential to Knodt’s attention, is illuminating for understanding the structures and boundaries of this semiosphere in particular: “a form of expression valid through the centuries” is, regardless of changes through modernization (“contemporary adaptation”), the “most original element of a people”. From all this, one can surmise that the category of the *genuine* or *authentic* is thus not really objective but, rather, something that is part of a larger strategy of exclusion. It forms one pole of a dualistic world view that is characterized by the exclusion of so-called *Gemeinschaftsfremde* (“aliens to the community”), a label whose membership could always be extended to a given segment of the population, including opponents of the government, non-heterosexual individuals, among others, who then become pariahs. In light of this, general remarks about the cultural significance of dance, in the context of theatre reviews of this sort, are best understood as inseparable from some form of racism. This is a form of discrimination that is, however, disguised and subtle rather than public and overt. Covert, racially biased distinctions tend to be hidden or rationalized with an explanation that society is willing to accept (for instance, referring to an “indigenous element” rather than “[*n*-word] exotic”). Thus, this particular theatre critique can be said to fit into a social climate that was clearly not (yet) too receptive to open rhetoric about war or the extermination of people. However, the seeds had already been planted.

## Case Study 2: The Binary Arteigen/Artfremd (“Racially Akin”/“Alien”) Distinction

The performances of the Indian Ballet took place from summer 1936 to spring 1938, a period in which the entire cultural sector was exposed to particular efforts to orient it towards the promotion of National Socialism. The aforementioned restrictions curtailing the feuilleton were augmented by the imposition of a biased form of evaluating art according to racial principles, in particular, ones grounded in the dichotomy of the *arteigen/artfremd* (“racially akin”/“alien”) or *entartet* (“degenerate”).

In January 1937, Wolfgang Willrich (1897–1948) published a polemic entitled *Säuberung des deutschen Kunsttempels. Eine kunstpolitische Kampfschrift zur Gesundung deutscher Kunst im Geiste nordischer Art* (The Purification of the German Temple of Art. An Artistic-Political Pamphlet for the Recovery of German Art in the Spirit of the Nordic Race) (Willrich 1937). Today, it is regarded as a model for the conceptualization and planning of the National Socialist exhibition “Degenerate Art” in Munich which took place the same year. Here, the author also unfolded the popular metaphor of the sick/healthy body of the people as a threatening scenario. Unlike in the post-First World War context, as described by Föllmer (2001), however, Willrich links the idea of national regeneration with a racist/*völkisch* orientation. In Part 4, he introduces the nature and aim of *German racial thought and its significance for German art* (Willrich 1937: 142–160), by claiming to have found the way out of the decadence caused by modernism and “cultural Bolshevism”. That a book like this could be accepted and debated during this time indicates, at least as early as 1937, that ideas regarding political deviance had begun to merge with ideas regarding one’s biological origin.

Interestingly, in the press reviews, Indians were not perceived as foreigners, but as something akin to distant relatives from whom one could learn a thing or two: “It was an evening of classical Indian music and dance. A model of national self-confidence that must be respected because it contains high art, folklore, and customs of which every people can and should be proud of (‑g. 1936).” The author here elevates the performance to a “classical” art form in accordance with the text of the programme booklet. In his mind, the performance had an almost model-like character in the sense of its possessing a specific identity. The normative expectation towards artists as bearers of national self-awareness is as present as the equation of art, folklore, and customs. Similarly, Albert Schneider wrote after a performance in Cologne:

“A world of artistic creation opens up, which, in the application of its means and forms, is completely different from that of a Western one and yet, this art is also deeply rooted in human thought and feeling and finds an expression which speaks of an intimate relationship of the cultic manifestations of a racially pure (*arteigen*) and strong people (*Volkstum*)” (Schneider 1936).^6^

These examples suggest that the public perceived the music and the dance troupe as more familiar than exotic, more akin than alien. Considering that more than a few theatre critics emphasized the exotic sounds and looks of the troupe, the suspicion arises that this kinship was emphasized for entirely different reasons than one might think at first.

## Excursus: Cultural Debates Around Music

The almost hymnal tributes to the Indian music and the dance troupe are situated within a discourse which centres around the value of music as *völkisch* art. Both in the wider musical life of this period and in contemporary musicology, there was a consensus that the Germans were the chosen *people of music*. In the nineteenth century, this legend was already widely accepted, and Germans emphasized their musical tradition as an expression of national identity and national character (Potter 1998: 11). Beyond the widespread nature of this belief, however, new developments in music were taking place that were primarily influenced by popular American music (jazz, swing) and thus often perceived as provocative. A clichéd politicization of German art and music had become an essential element of social discourse. “Undesirable” music became a political issue, defamed in the press with the same lines time and again (John 1994: 47). Influential conservative circles invoked an alleged threat to German culture based on their polemical concept of *musical *or *cultural Bolshevism* (Felbick 2015: 90).

After the mortification caused by the defeat in the First World War, a particular remedy was found in the thesis of the *world importance of German music*. Through their feeling of artistic and intellectual superiority, which hardly anyone in Germany disputed in the first half of the twentieth century, the injured national self-confidence of the Germans was able to recover. It was in this spirit that the *Kampfbund *was founded. Among its members was Alfred Heuss, the influential editor of the *Zeitschrift für Musik*, which served as an organ of the *Kampfbund* association, provided an influential platform for disseminating its aims (Felbick 2015: 90–91). It was also well-known for regularly publishing “smear campaigns by the German nationalist guardians of culture, especially the hate tirades against jazz” (Felbick 2015: 92). Against this backdrop, one particular mention of the Menaka troupe is especially striking. In an article published by Horst Büttner in the *Zeitschrift für Musik*, he begins with a section entitled *Praise of Romanticism*:

“Not only in the nineteenth century, but since the beginnings of German history, a distinctly Romantic trait of the German is evident: his urge towards the far and distant. This expresses itself artistically in the will and ability to participate spiritually in the art of foreign peoples and to incorporate it into his own design. This may sometimes lead to the danger of losing oneself; in general, however, this ability has considerably enriched German music, and the power of summarizing, which is often characteristic of German artists, has proved extraordinarily fruitful in many cases. However, the appeal of the exotic, that is, of the absolutely foreign and, precisely for that reason, appealing through its incomprehensibility, has been in most cases the least important reason” (Büttner 1937: 189).

After an appreciation of the music scene in the city of Leipzig, he follows up this somewhat cryptic remark on the appeal of the exotic with a review of the Indian programme. According to the author, the Menaka troupe’s distant and exotic charm—which critics mentioned and even praised in various other reviews—is at best a secondary cause for its continued success in Germany. In an extended reflection, he classifies the ballet’s successes as follows:

“This [its exotic appeal] partially provides the Indian Menaka Ballet with success in Germany today. But it is not the only reason why this ballet, with its brown dancers with excellent body control and instruments that are unfamiliar to us, has such an effect, even if it is an essential factor in the success of the troupe. The reasons are the specific areas of expressivity, to which facial expressions and music contribute, also speak to aspects of our people’s nature. In the case of the Menaka Ballet, this affinity is explicable since Menaka goes back to the ancient cult dances of her people. In other words, this is a ‘Romantic recourse to ancient folk tradition, comparable to the well-known efforts of the German Romantics’—and the Indians, like us, belong to the Aryan family of nations” (Büttner 1937: 190).

In contrast to the clichés of “foreign” music, which Knodt associates with jazz as part of a nationalistic *Zeitgeist* and which are also used by Büttner as a negative foil for *völkisch* musical productions, in speaking of the Menaka Ballet, both authors minimize the “exotic” in order to place the performance within a polarized worldview on the side of the familiar, which certainly required a certain amount of logical manoeuvring in both case. It also underlines the increased interest of both authors in developing a specific, politically motivated, interpretative agenda.

## The Menaka Ballet—Actualizing Knowledge About India?

Upon examining the texts about the Indian dance group and its orchestra, one particularly striking thing was the way in which they each emphasized the element of the *arteigen* and the field of authenticity. The reception of the Menaka Ballet was, thus, closely connected to a broader cultural knowledge about India that existed in German-speaking Europe long before the rise of National Socialism. Journalists and critics thus tended to integrate the performances into a broad framework of ideas concerning India that was based on a colonialist consumption of exotic spectacles as seen in (now infamous) human exhibitions which were popular in the past. These orientalist visions of India were characterized and fuelled by opulent costumes and bewitching, even if sometimes shrill, music. These stereotypes, in turn, were further perpetuated through the mass medium of cinema. Conversely, a different image emerges from the stereotype of the allegedly common origins of Germans and Indians. This article thus demonstrates that the motif of *Aryan* or *Aryan origins* was, at least during the time of the tour, part of an emotionally-charged discussion on the essence of art and its larger role in society. The linguistic and cultural semiotic analysis of the selected articles revealed the nodal points of the discourse—in particular, the semantic field of *authentic* or *genuine* as a normative category, in particular within the binary opposition of *arteigen/artfremd* (“racially akin”/“alien”) together with notion of the *entartet* (“degenerate”). Both served to show just how closely these discourses were related to a political attempt to create a master narrative of a racially homogenous and spiritually oriented social utopia. The issue of the contested semantic fields of authenticity and that of the *racially akin* versus the *degenerate *together work, in their own ways, to mark the intersections connecting art, society and politics. This is why the press reviews were able to so effectively participate in the construction of a specifically German social ideology based on autochthonous ideas that would eventually serve to distinguish Germany from other European nations. With the claim to have descended from the same origins as the Indians, German citizens could find grounds to separate themselves from their Jewish neighbours and other Europeans. The notion of a common origin for Germans and (Ancient) Indians was shared in learned circles and in a broad range of linguistic, cultural, philosophical and religious associations. In the increasingly aggressive climate of National Socialist (cultural) politics, the Indian artists were at the nodal point of various social strategies of differentiation. Presumably without realizing it, artists, spectators, and critics had become part of a cultural struggle that eventually paved the way for the state-sponsored degradation and dehumanization of countless people, eventually culminated in a ruthless policy of war and extermination.

## Acknowledgements

This article is based on a collaborative project with Markus Schlaffke (Weimar) which collects sources pertaining to the tour of the Indian Ballet Menaka in Germany and Europe. All newspaper articles cited can be found in the online archive at www.menaka-archive.org. Over the years, we have had countless discussions, and sometimes it is difficult to say who had which idea first. My sincere thanks thus go to him. Alan Daboin and Aaron French not only put this text into an elegant English form, but also removed countless intellectual inaccuracies, as did all those who read and decisively improved earlier versions of this text: Moritz Epple, Maria Framke, Eli Franco, Baijayanti Roy and Katharina Waldner. Moreover, two anonymous reviewers offered enormously helpful remarks. All deserve my sincerest thanks.

## Footnotes

1 Despite its great success with audiences, the organization and financing of the tour (with twelve musicians and dancers, and numerous costumes and instruments) was an expensive enterprise. For the financial balance sheet of the tour, see Schlaffke (2022: 78–83).

2 The concept of a Renaissance was not a descriptive, value-free comparison in the European and colonial historiography. This applies to Schlegel’s “Oriental Renaissance” (for example in Schwab 1950) as well as to the “Indian Renaissance,” which flourishing in Bengal in the nineteenth century and began with Ram Mohan Roy. I thank Eli Franco for the clarifying and helpful remarks. However, in the context of the Bayreuth circle around the family of Richard Wagner and Houston Stewart Chamberlain, there were in fact two “Indian Renaissances” in nineteenth-century Germany, namely that of German Romanticism and a second wave of interest in the Indian intellectual tradition in the wake of Arthur Schopenhauer (see Schwaderer forthcoming).

3 One of the members of the troupe, the versatile musician Ambika Charan Majumdar (1905–1943), seems to have been part of the independence movement. Instead of embarking the ship to India in 1938, he stayed in Germany and received a scholarship from the University of Königsberg (Kaliningrad) until 1939. His name appears in a document of the intelligence bureau of the colonial Government of India: ‘Notes on suspect civilian Indians on the Continent of Europe.’ Nationalist leader Subhas Chandra Bose (1897–1945), founder of the *Azad Hind* [Free India] organizations, seems to have known Majumdar’s family from Calcutta, and the musician set up *Namaste Hind*, broadcasts on Indian culture for the *Reichs-Rundfunk-Gesellschaft* (Reich Broadcasting Corporation) (Kuhlmann 2003: 172). He seems to have been among the victims of the bombing of Dresden in February 1945 (Bose et al. 1973: 132). For more details, see Kanhai (2020).

4 Leonie Dotzler-Möllering (1899–1984) initially entered Mary Wigman’s first dance class in Dresden. However, she soon had to end her career as a dancer and joined the *Dresdner Neuesten Nachrichten* as a trainee in 1922. From 1928 onwards, she worked as an editor in the feuilleton with a focus on operetta, film, and dance criticism, as well as concert and theatre (see Frank-Manuel Peter undated) Her interview with Menaka and her theatre criticism placed the performance in a contemporary dance context, which is quite unlike how many of her colleagues approached it.

5 The *Coburger Nationalzeitung* (Coburg National Newspaper) was founded in 1930, one year after the NSDAP had become the strongest faction in the Coburg city council, expanding on the National Socialist weekly *Weckruf*, by Coburg NSDAP chairman Franz Schwede (1888–1960), who was elected the first Nazi mayor of a German city in 1931. The newspaper so successfully combined National Socialist propaganda and reports from everyday life in the region that its main competitor, the *Coburger Zeitung*, ceased publication in 1935. On the initiative of Gauleiter Hans Schemm, the *Coburger Nationalzeitung* became affiliated to the Bayerische Ostmark GmbH in Bayreuth in 1934 (see Anonymous undated: *Zur Coburger Nationalzeitung*).

6 Albert Schneider (1905–1985) was a music teacher, dialect poet and composer from Cologne. After attending the conservatory, where he passed the pedagogical exams for piano and voice, he worked as a music teacher and independent artist in film and radio. He also began to compose and conducted operas staged by the *Bühnen-Volksbund* (“Stage Folk League”) as music director. According to his own statements, he joined the NSDAP in March 1933. During the Third Reich, he was a freelancer and music consultant for the *Kölner Lokal-Anzeiger*, later called *Der neue Tag.* After this newspaper was banned, he worked for the *Kölnische Volkszeitung*, which was discontinued in 1941 (State Archive of North Rhine Westphalia, Sgn. Best 1298).
